# Anti‐apoptotic effects of human gingival mesenchymal stromal cells on polymorphonuclear leucocytes

**DOI:** 10.1111/odi.13768

**Published:** 2021-01-15

**Authors:** Alice Blufstein, Christian Behm, Barbara Kubin, Johannes Gahn, Andreas Moritz, Xiaohui Rausch‐Fan, Oleh Andrukhov

**Affiliations:** ^1^ Department of Conservative Dentistry and Periodontology University Clinic of Dentistry Medical University of Vienna Vienna Austria

**Keywords:** gingiva, immunomodulation, mesenchymal stromal cells, periodontitis, polymorphonuclear leucocytes

## Abstract

**Objectives:**

Polymorphonuclear leucocytes (PMNs) constitute the first line of host defence and are crucial in maintaining periodontal health. Their survival and function are modulated by mesenchymal stromal cells (MSCs) from different origin. Gingival MSCs (GMSCs) play an important role in maintaining oral health and in the initial inflammatory response. The present study aimed to investigate the effects of GMSCs on PMNs apoptosis and reactive oxygen species (ROS) production.

**Methods:**

PMNs were either directly incubated with untreated, interleukin (IL)‐1β‐ or tumour necrosis factor (TNF)‐α‐treated GMSCs or stimulated with their conditioned media. Resulting ROS production was evaluated by dichlorofluorescin diacetate staining, whereas PMNs apoptosis was assessed by Annexin V staining, followed by flow cytometry analysis.

**Results:**

While conditioned media of untreated and TNF‐α‐treated GMSCs did not affect apoptosis of PMNs, it was significantly delayed by conditioned media of GMSCs treated with IL‐1β. In direct co‐culture, GMSCs exerted anti‐apoptotic effects on PMNs independently of the previous stimulation. However, the strongest impact was observed by IL‐1β‐treated GMSCs. ROS production of PMNs was not influenced by GMSCs or their conditioned media.

**Conclusion:**

This study demonstrates for the first time the immunomodulatory properties of GMSCs towards PMNs, revealing that IL‐1β enhances anti‐apoptotic effects of GMSCs.

## 1 INTRODUCTION

Periodontitis is an utterly complex disease modulated by multiple factors and characterized by a chronic course (Slots, 2017). This widely spread inflammatory disease affects all components of the periodontium, namely gingiva, periodontal ligament, alveolar bone and cementum, and may lead to tooth loss in severe cases (Hassell, 1993; Petersen & Baehni, 2012). Periodontitis is initiated by a shift of oral microbiota to a dysbiotic character and the thereby emerging activity of the innate and acquired immune system, which collaterally damages periodontal tissues (Meyle & Chapple, 2015). Different types of resident and immune cells, as well as numerous cytokines, chemokines and growth factors, are involved in the complex mechanisms of the immune response during periodontitis (Cekici et al., 2014).

Surrounding the neck of the tooth, the gingiva not only acts as a mechanical barrier against invading pathogens, but also plays a crucial role in the innate immune response (Fujita et al., 2018). Thereby, gingival mesenchymal stromal cells (GMSCs) are of pivotal importance. Fulfilling the criteria of mesenchymal stromal cells (MSCs), GMSCs are characterized by their strong immunomodulatory properties (Dominici et al., 2006; Fawzy El‐Sayed & Dörfer, 2016). GMSCs have been observed to express pro‐inflammatory cytokines in response to different inflammatory stimuli, such as Toll‐like receptor agonists (Agarwal et al., 1995; Kent et al., 1998; Palmqvist et al., 2008). Furthermore, interleukin (IL)‐1β and tumour necrosis factor (TNF)‐α induce the expression of chemotactic factors such as IL‐8 by GMSCs, which is a potent chemoattractant for polymorphonuclear leucocytes (PMNs) (Takashiba et al., 1992).

As the first line of host defence, PMNs are key players of the innate immune system (Kobayashi & DeLeo, 2009). Entering the sites of inflammation via transendothelial migration, they can apply a variety of mechanisms to eliminate invading pathogens, such as production of reactive oxygen species (ROS), phagocytosis and neutrophil extracellular trap (NET) formation (Borregaard, 2010; Choi et al., 2009; Hirschfeld et al., 2015; Lee et al., 2003; Nguyen et al., 2017). Moreover, programmed cell death of PMNs constitutes an essential factor in the resolution of inflammation, which can be modulated by inflammatory stimuli such as IL‐1β and TNF‐α (Akgul et al., 2001; Fox et al., 2010).

Since PMNs represent more than 95% of leucocytes recruited into the gingival sulcus, their number, distribution and function is of major importance for periodontal health (Delima & Van Dyke, 2003; Hajishengallis, 2015). Although the immunomodulatory properties of MSCs on PMNs have been the subject of several studies, which observed prolongation of their survival and function, there is only little evidence about the influence exerted by periodontium‐derived MSCs (Brandau et al., 2010; Cassatella et al., 2011). In particular, there are no data concerning the effect of GMSCs on PMNs. Therefore, the aim of this study was to assess the impact of GMSCs on apoptosis and ROS production of PMNs under physiological and inflammatory conditions simulated with IL‐1β and TNF‐α. In order to evaluate a dependence on direct cell‐to‐cell or soluble factors, two different approaches were adopted: direct co‐culture and conditioned media of GMSCs.

## 2 MATERIALS AND METHODS

### 2.1 Ethical considerations

The protocol for isolation of hGMSCs and PMNs, as well as the study protocol, was approved by the ethics committee of the Medical University of Vienna (ethical approval number: 1694/2015, revised in 2019). The Declaration of Helsinki and the “Good Scientific Practice” guidelines of the Medical University of Vienna were followed throughout all experiments.

### 2.2 Isolation and cultivation of primary human gingival mesenchymal stromal cells

hGMSCs were isolated from gingival tissue attached to extracted third molars of eight individuals. The donors were Caucasian non‐smokers (four females, four males) between 18 and 24 years with no systemic or oral diseases, who underwent tooth extraction due to orthodontic indications. All patients gave their informed written consent prior to the extraction. After gently washing the teeth with phosphate buffered saline, gingival tissue was cut off using a scalpel. The tissue fragments were placed into Petri dishes containing Dulbecco's modified Eagle's medium (DMEM; Sigma‐Aldrich.) supplemented with 10% foetal bovine serum (FBS; Gibco) and 50 µg/ml penicillin and 100 U/ml streptomycin (P/S; Gibco), and minced into small pieces. Petri dishes were incubated under humidified conditions at 5% CO_2_ and 37°C, and after outgrowth of cells (~7 days), they were transferred to cultural flasks for expansion (~21 days). In order to evaluate mesenchymal stromal cell character, cells were detached with Accutase^®^ (Stemcell Technologies) and stained for characteristic mesenchymal surface markers CD29, CD73, CD90, CD105 and CD146, as well as negative expression of hematopoietic surface markers CD31, CD34 and CD45 (all antibodies from Thermo Fisher Scientific), followed by flow cytometry analysis (Andrukhov et al., 2016). Furthermore, tri‐lineage differentiation of hGMSCs was confirmed. Cells were used in passage 4 to 6 in the following experiments and all donors were individually evaluated.

### 2.3 Isolation of human primary polymorphonuclear leucocytes

Whole blood was collected from one healthy volunteer using Vacuette Safety G21 and lithium heparin‐coated Vacuette tubes (both Greiner Bio One International GmbH). Isolation of primary PMNs was executed as described by Oh et al. (2008). Briefly, 5 ml of whole blood was carefully overlaid onto 5 ml Lympholyte‐poly cell separation media (Cedarlane) and centrifuged at 2,000 revolutions per minute and 20°C for 35 min. While the upper three of the six resulting layers were disposed, the layers containing PMNs and separation media were collected and washed with Hank's Balanced Salt solution (HBSS; Gibco™). After centrifugation for 10 min, supernatants were removed and the pellet was resuspended with 2 ml Red Blood Cell Lysis buffer (Santa Cruz). Subsequently, cells were centrifuged for 5 min and the lysing process was repeated. The supernatants were discarded, and the pellet was resuspended with 10 ml HBSS and centrifuged for 5 min, followed by resuspension of the PMNs pellet with HBSS containing Ca^2+^ and Mg^2+^. After confirmation of PMNs purity by flow cytometry analysis of surface markers CD16 (eBioCB16 (CB16); FITC, eBioscience) and CD62L (MEL‐14; FITC, eBioscience™), cells were immediately used for experiments.

### 2.4 Treatment of PMNs with conditioned media of hGMSCs

hGMSCs of eight donors were seeded in 24‐well plates at a density of 5 × 10^4^ cells in 0.5 ml DMEM supplemented with 10% FBS and 1% P/S and incubated for 24 hr. Subsequently, cells were washed with PBS and stimulated with serum‐free DMEM containing either 5 ng/ml IL‐1β, 10 ng/ml TNF‐α (both PeproTech) or no inflammatory stimuli. These concentrations were selected based on previous studies (Andrukhov et al., 2020; Behm et al., 2020). At the completion of 24‐hr incubation time, stimulation solutions were discarded and cells were washed with PBS. Afterwards, hGMSCs were incubated with Roswell Park Memorial Institute media 1,640 (RPMI; Sigma‐Aldrich) for 24 hr to collect proteins excreted in response to inflammatory stimuli and conditioned media were collected. Falcon^®^ Round‐Bottom Polystyrene tubes (5 ml; Sarstedt.) were used to incubate 1 × 10^6^ PMNs/ml with conditioned media in different dilutions and for different time periods, which differed depending on the subsequent analysis and were selected based on preliminary experiments. For evaluation of apoptosis, PMNs were incubated with conditioned media diluted 1:1 with FBS‐free RPMI for 3 and 24 hr (37°C, 5% CO_2_). To evaluate ROS production, conditioned media were diluted 1:10 with FBS‐free RPMI and PMNs were stimulated at 37°C and 5% CO2, which was stopped after 15 min by putting the cells on ice. PMNs treated with FBS‐free RPMI served as negative control group, whereas PMNs stimulated with FBS‐free RPMI containing 100 ng/ml phorbol myristate acetate (PMA; Sigma‐Aldrich) were considered as positive control. Stimulation of PMNs was always performed in the presence of 1 µg/ml ultrapure *Porphyromonas gingivalis* lipopolysaccharide (up*Pg*LPS; Invivogen).

### 2.5 Direct co‐culture of hGMSCs and PMNs

hGMSCs of eight donors were seeded in 24‐well plates at a density of 5 × 10^4^ cells in 0.5 ml DMEM per well containing 10% FBS and 1% P/S. After incubation for 24 hr, media were discarded, and cells were washed with PBS and stimulated with FBS‐free DMEM containing 5 ng/ml IL‐1β, 10 ng/ml TNF‐α or no inflammatory stimuli for 24 hr. After washing the cells twice with FBS‐free RPMI for 10 min, hGMSCs were co‐cultured with 1 × 10^6^ freshly isolated PMNs in FBS‐free RPMI. PMNs incubated in monoculture with FBS‐free RPMI were considered as negative control group. Mono‐cultured PMNs treated with 100 ng/ml PMA in FBS‐free RPMI were utilized as positive control. Treatment of PMNs was always performed in the presence of 1 µg/ml up*Pg*LPS. Since PMNs do not adhere, they were easily collected after 15 min for ROS measurement and after 3 and 24 hr for analysis of apoptosis. The time points for the apoptosis measurements were selected based on preliminary experiments and represent two important time points, namely early apoptosis (~20%–40% apoptotic PMNs after 3 hr) and late apoptosis (~90% apoptotic PMNs after 24 hr).

### 2.6 Flow cytometry analysis

Apoptosis of PMNs was assessed by Annexin V staining. For this purpose, the eBioscience™ Annexin V Apoptosis Detection Kit FITC (Thermo Fisher) was used. ROS production by PMNs was evaluated by dichlorofluorescin diacetate (DCFDA) staining, which was facilitated by the Abcam's DCFDA Cellular ROS Detection Assay Kit (Abcam). Both kits were utilized according to the manufacturer's instructions. Annexin V‐ and DCFDA‐positive cells were analysed with a FACSCalibur flow cytometer (Becton Dickinson). Fluorescence was excited with an argon laser at 488 nm for a total number of 10,000 cells. For the analysis, all cells were included without additional gating. The percentage of Annexin V and DCFDA positive cells was determined using CellQuest 3.3 software (Becton Dickinson).

### 2.7 Statistical analyses

Statistical analyses were performed with SPSS 24.0 (IBM). Kolmogorov‐Smirnov test was applied in order to verify normality. Differences between groups were assessed by ANOVA for paired measures, followed by *t*‐test for pairwise comparison. *p*‐values <.05 were considered as statistically significant. Data are presented as mean values ± standard error of the mean (*SEM*) obtained from eight independent experiments performed in duplicates using GMSCs from eight different individuals.

## 3 RESULTS

### 3.1 Surface marker analysis of GMSCs and PMNs

Surface marker expression of GMSCs used in this study was analysed by flow cytometry and is displayed in Table 1, showing positive expression of MSCs markers and negative expression of hematopoietic stem cell markers. More than 95% of cells were positively stained for the tested MSCs markers, except for CD146, which is commonly observed (Fawzy El‐Sayed & Dörfer, 2016). The percentage of CD31‐, CD34‐ and CD45‐positive cells was below 2.5%. Representative dot plots of each surface marker are presented in Figure S1.

**TABLE 1:** Surface marker expression of GMSCs

	Mesenchymal stromal cell markers					Hematopoietic stem cell markers		
	CD29	CD73	CD90	CD105	CD146	CD31	CD34	CD45
% of positive cells	98.49	97.99	95.69	97.79	75.62	0.31	1.84	2.49
*SEM*	0.41	0.35	0.64	1.15	8.74	0.14	0.38	0.58

Note

Primary GMSCs (*n* = 8) were stained for mesenchymal stromal cell markers CD29, CD73, CD90, CD105 and CD146, as well as hematopoietic stem cell markers CD31, CD34 and CD45, and analysed by flow cytometry. This shows the mean percentage of positively stained cells ±*SEM*.

Figure S2 shows representative dot plots of characteristic PMNs surface markers analysed with flow cytometry. As can be seen, more than 96% of PMNs were positively stained with CD11, CD16 and CD66.

### 3.2 Effect of GMSCs on PMNs apoptosis

**FIGURE 1:**
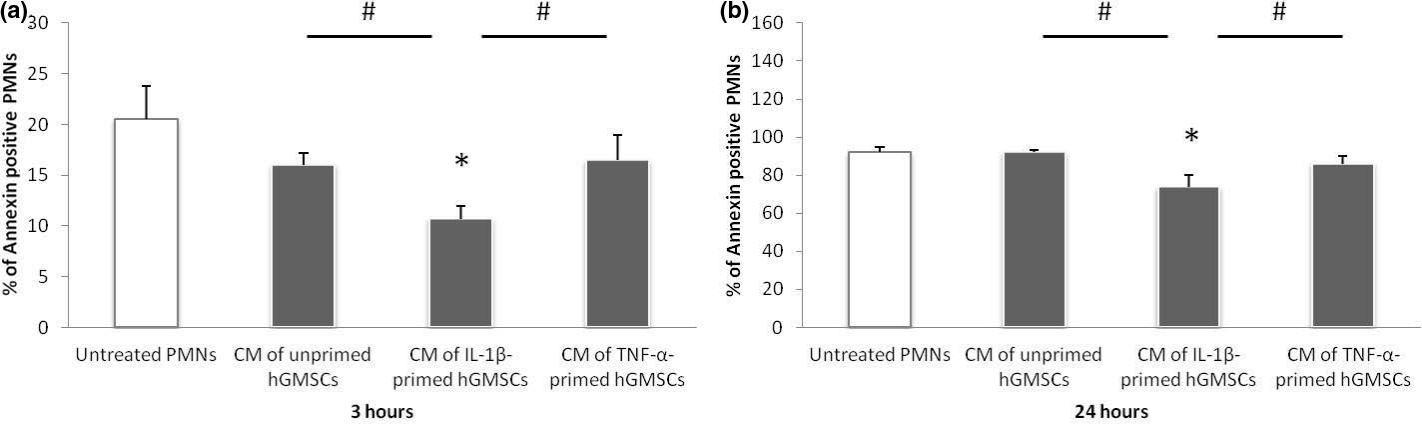
Effect of conditioned media obtained from GMSCs on apoptosis of PMNs. Primary GMSCs (*n* = 8) were incubated with IL‐1β (5 ng/ml), TNF‐α (10 ng/ml) or without inflammatory stimuli for 24 hr. Conditioned media (CM) were diluted 1:1 with RPMI and utilized for treatment of PMNs at a ratio of 1 × 10^6^ cells/ml for 3 (a) and 24 (b) hours. PMNs incubated in RPMI served as control group. Incubation of PMNs was performed in the presence of 1 µg/ml up*Pg*LPS. PMNs apoptosis was analysed by staining with Annexin V Apoptosis Detection Kit FITC and subsequent flow cytometry. The *y*‐axes represent the percentage of Annexin V positive cells. Data are presented as mean ± standard error of the mean (*SEM*). *Significant decrease compared to untreated PMNs, *p* < .05, # Significant difference between groups as indicated, *p* < .05

Figure 1 illustrates the effect of conditioned media, obtained from GMSCs treated with either 5 ng/ml IL‐1β, 10 ng/ml TNF‐α or no inflammatory stimuli, on the apoptosis of PMNs after 3 (Figure 1a) and 24 (Figure 1b) hours. Representative dot plots of Annexin V‐stained untreated PMNs are shown in Figure S3.

The percentage of Annexin V positive PMNs after 3 hr was about 20%. Although conditioned media of untreated GMSCs decreased PMNs apoptosis, no significant difference could be observed. Similarly, incubation of PMNs with conditioned media of TNF‐α‐treated GMSCs had no significant influence on their apoptosis. In contrast, the percentage of Annexin V positive PMNs was significantly decreased after 3‐hr incubation with conditioned media of GMSCs treated with IL‐1β in comparison with all other groups.

After 24 hr of incubation with RPMI, more than 90% of PMNs were positively stained for Annexin V. Treatment of PMNs with conditioned media of GMSCs for 24 hr revealed the same tendency as after 3 hr. While conditioned media of untreated and TNF‐α‐treated GMSCs did not affect PMNs apoptosis, it was significantly decreased in the presence of conditioned media obtained from GMSCs treated with IL‐1β. This effect was observed in comparison to all tested groups.

**FIGURE 2:**
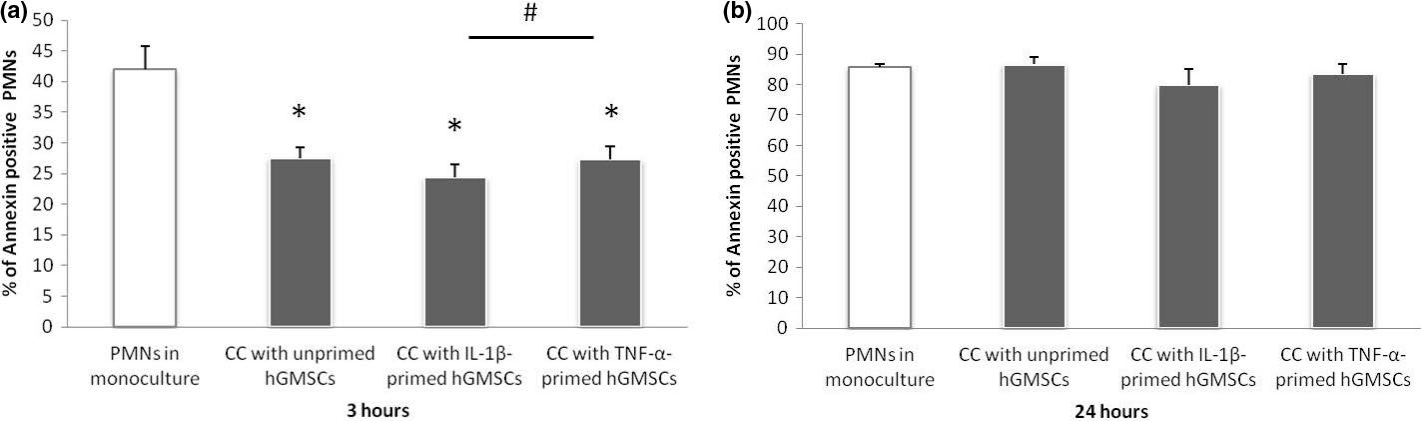
Effect of GMSCs on apoptosis of PMNs in direct co‐culture. Primary GMSCs (*n* = 8) were incubated with IL‐1β (5 ng/ml), TNF‐α (10 ng/ml) or without inflammatory stimuli for 24 hr. Stimulation media were removed, and GMSCs were incubated with 1 × 10^6^ PMNs in RPMI for 3 (a) and 24 (b) hours. PMNs incubated in monoculture with RPMI served as control group. Incubation of PMNs was performed in the presence of 1 µg/ml up*Pg*LPS. PMNs apoptosis was analysed by staining with Annexin V Apoptosis Detection Kit FITC and subsequent flow cytometry. The *y*‐axes represent the percentage of Annexin V‐positive cells. Data are presented as mean ± *SEM*. *Significant decrease compared to untreated PMNs, *p* < .05, #Significant difference between groups as indicated, *p* < .05

The percentage of Annexin V‐positive PMNs after co‐culture with untreated, 5 ng/ml IL‐1β‐ and 10 ng/ml TNF‐α‐treated GMSCs for 3 (A) and 24 (B) hours is shown in Figure 2.

About 40% of PMNs in monoculture were stained positively for Annexin V after 3 hr. Co‐culture with GMSCs significantly diminished PMNs apoptosis, which was independent from the inflammatory stimuli. The strongest anti‐apoptotic effect was observed by IL‐1β‐treated GMSCs, which was significantly more pronounced in comparison with GMSCs stimulated with TNF‐α.

In contrast to the findings after 3 hr, apoptosis of PMNs was not affected by co‐culture with untreated and treated GMSCs for 24 hr.

### 3.3 Effect of GMSCs on ROS production by PMNs

**FIGURE 3:**
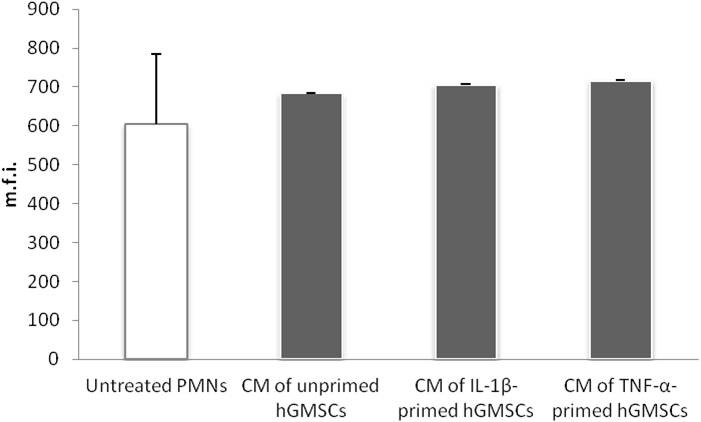
Effect of conditioned media obtained from GMSCs on ROS production by PMNs. Primary GMSCs (*n* = 8) were incubated with IL‐1β (5 ng/ml), TNF‐α (10 ng/ml) or without inflammatory stimuli for 24 hr. Conditioned media were diluted 1:10 with RPMI and utilized for treatment of PMNs at a ratio of 1 × 10^6^ cells/ml for 15 min. PMNs incubated with RPMI or PMA (100 ng/ml; >95% DCFDA positive cells, data not shown) served as negative and positive control group, respectively. Incubation of PMNs was performed in the presence of 1 µg/ml up*Pg*LPS. ROS production was analysed by staining PMNs with DCFDA Cellular ROS Detection Assay Kit and subsequent flow cytometry. The *y*‐axis represents the mean fluorescent intensity (m.f.i.) of DCFDA‐positive PMNs. Data are presented as mean ± *SEM*

The resulting ROS production of PMNs after 15‐min incubation with conditioned media of untreated, IL‐1β‐ (5 ng/ml) and TNF‐α‐treated (10 ng/ml) GMSCs is presented in Figure 3. Representative dot plots of DCFDA‐stained untreated PMNs are illustrated in Figure S4 Conditioned media of both untreated and treated GMSCs had no influence on the mean fluorescent intensity (m.f.i.) of DCFDA‐positive PMNs after 15 min in comparison with PMNs incubated in RPMI.

**FIGURE 4:**
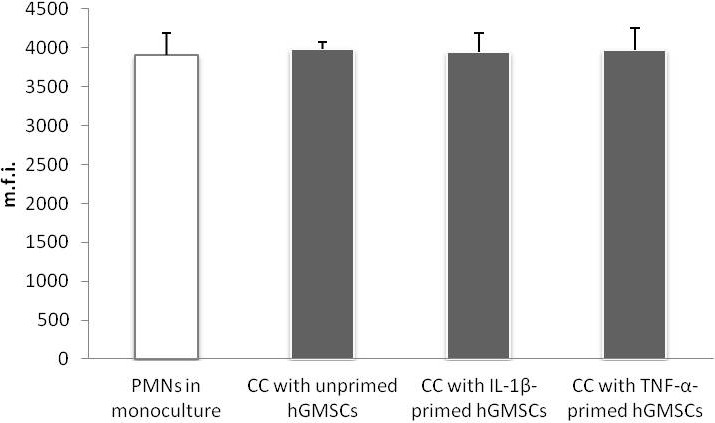
Effect of GMSCs on ROS production by PMNs in direct co‐culture. Primary GMSCs (*n* = 8) were incubated with IL‐1β (5 ng/ml), TNF‐α 10 ng/ml) or without inflammatory stimuli for 24 hr. Stimulation media were removed, and GMSCs were incubated with 1 × 10^6^ PMNs in RPMI for 15 min. PMNs incubated in monoculture with RPMI or PMA (100 ng/ml; >95% DCFDA‐positive cells, data not shown) served as negative and positive control group, respectively. Incubation of PMNs was performed in the presence of 1 µg/ml up*Pg*LPS. ROS production was analysed by staining PMNs with DCFDA Cellular ROS Detection Assay Kit and subsequent flow cytometry. The *y*‐axis represents the m.f.i. of DCFDA positive PMNs. Data are presented as mean ± *SEM*

Figure 4 visualizes the effects of 15 min direct co‐culture with GMSCs previously treated with IL‐1β (5 ng/ml), TNF‐α (10 ng/ml) or no inflammatory stimuli. Similarly to GMSCs conditioned media, direct co‐culture of untreated and treated GMSCs with PMNs had no effect on the m.f.i. of DCFDA‐positive PMNs.

## 4 DISCUSSION

PMNs are not only the most abundant leucocytes in the blood stream, but also in the gingival sulcus (Sima & Glogauer, 2014). Their functionality is a vital factor for maintaining periodontal health. Contrary to other immune cells like T cells and macrophages, the immunomodulatory properties of periodontal MSCs on PMNs are poorly investigated (Andrukhov et al., 2019). Thus, the present study aimed to investigate for the first time the immunomodulatory properties of GMSCs on PMNs.

Two different approaches were pursued in order to elucidate the influence of GMSCs on PMNs apoptosis and ROS production. Firstly, conditioned media of untreated and pretreated GMSCs were obtained to evaluate the importance of soluble factors. Secondly, PMNs were directly co‐cultured with untreated and pretreated GMSCs to assess the impact of direct cell‐to‐cell contact. As PMNs dominate the initial immune response, treatment of GMSCs was performed with IL‐1β and TNF‐α, which are known to occur in a very early phase of inflammation and are crucially involved in periodontal tissue destruction (Kayal, 2013; Tawfig, 2016; Zhang et al., 2017). These cytokines are not only expressed by various immune cells, but also by MSCs from different origin (Dinarello, 2018; Hwang et al., 2009; Li et al., 2013; Šedý et al., 2015). The applied concentrations of IL‐1β (5 ng/ml) and TNF‐α (10 ng/ml) were selected based on previous studies. Behm et al. (2020) show the dose‐dependent response of MSCs derived from periodontal ligament in the supplementary material of their study. Moreover, Andrukhov et al. (2020) used the same concentrations in their study with GMSCs. In addition, 5 ng/ml IL‐1β reflects the concentration in gingival crevicular fluid of periodontitis patients (Engebretson et al., 2004). Furthermore, treatment of PMNs with conditioned media and direct co‐culture with GMSCs was performed in the presence of up*Pg*LPS to simulate bacterial invasion in the initial phase of periodontitis.

Although PMNs are considered to be rather short‐living cells (7–12 hr in vivo), their lifespan can expand to 48 hr during inflammation (Fox et al., 2010; Kennedy & Deleo, 2009). Apoptosis of PMNs and subsequent elimination by macrophages are indispensable for the resolution of inflammation (Akgul et al., 2001; Fox et al., 2010). Furthermore, the production of PMNs in the bone marrow is mainly regulated by their rate of apoptosis (Borregaard, 2010). In the present study, apoptosis of PMNs was differently triggered by soluble factors excreted by GMSCs, depending on their previous stimulation. While conditioned media collected from untreated and TNF‐α‐treated GMSCs did not affect PMNs apoptosis, the soluble factors expressed by IL‐1β‐treated GMSCs showed a significant anti‐apoptotic effect. This observation was made after both, 3 and 24 hr.

A similar tendency was observed in PMNs treated in direct co‐culture with GMSCs. In this case, PMNs apoptosis was significantly inhibited even by contact with un‐primed GMSCs for 3 hr. This effect was increased by previous stimulation with IL‐1β, which led to a significantly stronger anti‐apoptotic effect compared to untreated and TNF‐α‐treated GMSCs. According to the experiments with direct co‐culture, cell‐to‐cell interaction between GMSCs and PMNs might also influence PMNs apoptosis, in addition to soluble factors. A potential mechanism might be related to the apoptosis‐mediating Fas/Fas ligand pathway (Liles et al., 1996). However, the nature and physiological role of direct cell‐to‐cell interaction should be further established.

The results of our study revealed substantial differences in the anti‐apoptotic effects of GMSCs, depending on their previous stimulation. The stronger anti‐apoptotic effects of IL‐1β‐primed GMSCs could be explained by the resulting IL‐6 expression. Both IL‐1β and TNF‐α have been shown to enhance the IL‐6 expression in GMSCs. However, 5 ng/ml IL‐1β induces five times higher IL‐6 protein production in GMSCs than 10 ng/ml TNF‐α (Andrukhov et al., 2020). IL‐6 has been shown to be highly effective in delaying PMNs apoptosis (Biffl et al., 1996). Moreover, it is considered to be one of the factors responsible for the anti‐apoptotic effects of bone marrow‐derived MSCs (Cassatella et al., 2011). Although this is a possible explanation for our findings, further studies are necessary to verify these assumptions with mechanistic experiments.

Our findings are only partially in consistence with studies conducted on extra‐oral or other periodontal MSCs. Raffaghello et al. (2008) observed that bone marrow‐derived MSCs inhibit PMNs apoptosis in both direct and indirect co‐culture. Similar effects were reported by Cassatella et al. in Toll‐like receptor activated bone marrow‐derived MSCs (Cassatella et al., 2011). In studies conducted with human periodontal ligament stromal cells (hPDLSCs), PMNs apoptosis was also delayed independently from direct cell contact (Cianci et al., 2016; Wang et al., 2017). However, all of these studies applied indirect co‐culture models with transwell inserts to assess the effects of soluble factors, and thus, the comparability is aggravated.

Another aspect of this study was to investigate the impact of GMSCs on the production of ROS by PMNs. These partially reduced metabolites of oxygen with oxidizing capabilities are produced as a result of PMNs activation and subsequent induction of nicotinamide adenine dinucleotide phosphate oxidase. They can be either released into phagosomes or the extracellular environment in order to eliminate invading pathogens (Lee et al., 2003; Mittal et al., 2014; Nguyen et al., 2017). Furthermore, ROS trigger the formation of NET, which is another lethal weapon of PMNs (Björnsdottir et al., 2015). The results of this study revealed that ROS production by PMNs is neither increased by soluble factors excreted by GMSCs, nor by direct cell‐to‐cell contact with GMSCs. This observation is somewhat surprising, as the apoptosis rate of PMNs increases with their ROS production (Matés & Sánchez‐Jiménez, 2000). Thus, GMSCs seem to disrupt the coupling between PMNs ROS production and apoptosis, and increase their lifespan without diminishing ROS production.

In our experimental conditions, we did not detect any effect of GMSCs on PMNs ROS production. However, ROS produced by PMNs might have had an impact on GMSCs in the co‐culture experiments. Numerous studies have investigated the influence of oxidative stress on different MSCs, revealing an impairment of their self‐renewal, differentiation capacity and proliferation (Alves et al., 2010; Choo et al., 2014; Ko et al., 2012; Meagher et al., 1988). In addition, ROS has been shown to inhibit the osteogenic differentiation of MSCs, as well as their immunomodulatory potential (Denu & Hematti, 2016). Obviously, there is a complex reciprocal interaction between GMSCs and PMNs during co‐culture, which depends on various factors, such as the ratio between the cells, incubation time and other experimental conditions. Therefore, to fully understand the interaction between these two cell types, future studies should focus on testing other GMSCs:PMNs ratios or conditioned media production protocols.

It is noteworthy that the percentage of Annexin and DCFDA‐positive untreated PMNs clearly differed between the different experiments. This could possibly be explained by the fact that different series of experiments were performed for conditioned media and direct co‐culture. However, apoptosis and ROS measurement was performed on the same day of the respective treatment modality. Thus, our conclusion that GMSCs delays apoptosis without affecting ROS production is still valid.

Another aspect that should be considered is the exposure of GMSCs to inflammatory stimuli, which might influence their differentiation potential and function. However, the stimulation time was kept rather short. Furthermore, all stimulation solutions were prepared in serum‐free media, which do not support cell differentiation and proliferation.

Our data could be of major clinical relevance, because it has been shown that GMSCs increase PMNs survival. On the one hand, this indicates increased defence capacity of PMNs. On the other hand, delayed PMNs apoptosis is associated with increased tissue destruction due to a prolonged period of ROS production (Sima & Glogauer, 2014).

However, although advanced co‐culture models were used in the present study, it is limited by its in vitro character. Another limitation is that PMNs were isolated from one donor; however, this was necessary to focus solely on the effects of GMSCs and exclude the inter‐individual variability of PMNs. Since GMSCs were only isolated from healthy donors, this study could be extended by including individuals with periodontitis. Future studies should further not only focus on other PMNs functions, but also investigate the immunomodulatory properties of GMSCs on other immune cells, such as dendritic cells or natural killer cells. Another interesting and novel aspect would be to investigate the effects of periodontal MSCs on oral PMNs, which have been shown to have a rather hyperactive character and an enhanced lifespan (Moonen et al., 2019). Oral PMNs migrate to the gingival crevice through gingival tissue and their interaction with GMSCs could partially underlie their extended lifespan.

Summarizing, this study demonstrated the immunomodulatory properties of GMSCs towards PMNs for the first time. In particular, GMSCs have been shown to prolong PMNs survival, which might be an important factor in the initial phase of periodontitis. Further studies are needed to elucidate the immunomodulatory properties of periodontium‐derived MSCs and their role for periodontal health.

## CONFLICT OF INTEREST

The authors declare no conflict of interest.

## AUTHOR CONTRIBUTIONS

**Alice Blufstein:** Conceptualization; Formal analysis; Funding acquisition; Investigation; Methodology; Writing‐original draft. **Christian Behm:** Validation; Writing‐review & editing. **Barbara Kubin:** Investigation; Methodology. **Johannes Gahn:** Data curation; Validation. **Andreas Moritz:** Resources; Writing‐review & editing. **Xiaohui Rausch‐Fan:** Funding acquisition; Resources; Writing‐review & editing. **Oleh Andrukhov:** Conceptualization; Funding acquisition; Methodology; Project administration; Software; Supervision; Writing‐original draft.

### PEER REVIEW

The peer review history for this article is available at https://publons.com/publon/10.1111/odi.13768.

## Supporting information

Figures S1‐S4

## Data Availability

The data sets used and/or analysed during the current study are available from the corresponding author on reasonable request.
