# Supplementary material for: Anti‐apoptotic effects of human gingival mesenchymal stromal cells on polymorphonuclear leucocytes
Source: Oral Dis. 2021 Jan 15;28(3):777–85. doi: 10.1111/odi.13768 (PMC9290793; doi:10.1111/odi.13768)
Supplement: Supplementary file 1 — Figures S1‐S4 [file ODI-28-777-s001.docx]

**Supporting Information**

# Anti-apoptotic effects of human gingival mesenchymal stromal cells on polymorphonuclear leukocytes

# Running Title: Immunomodulation of neutrophils by gingival MSCs

Alice Blufstein^1^, Christian Behm^1^, Barbara Kubin^1^, Johannes Gahn^1^, Andreas Moritz ^1^, Xiaohui Rausch-Fan ^1^, Oleh Andrukhov^1^

^1^University Clinic of Dentistry, Division of Conservative Dentistry and Periodontology, Medial University of Vienna, Vienna, Austria

**^*^Corresponding author:**

Andrukhov Oleh

[oleh.andrukhov@meduniwien.ac.at](mailto:oleh.andrukhov@meduniwien.ac.at)


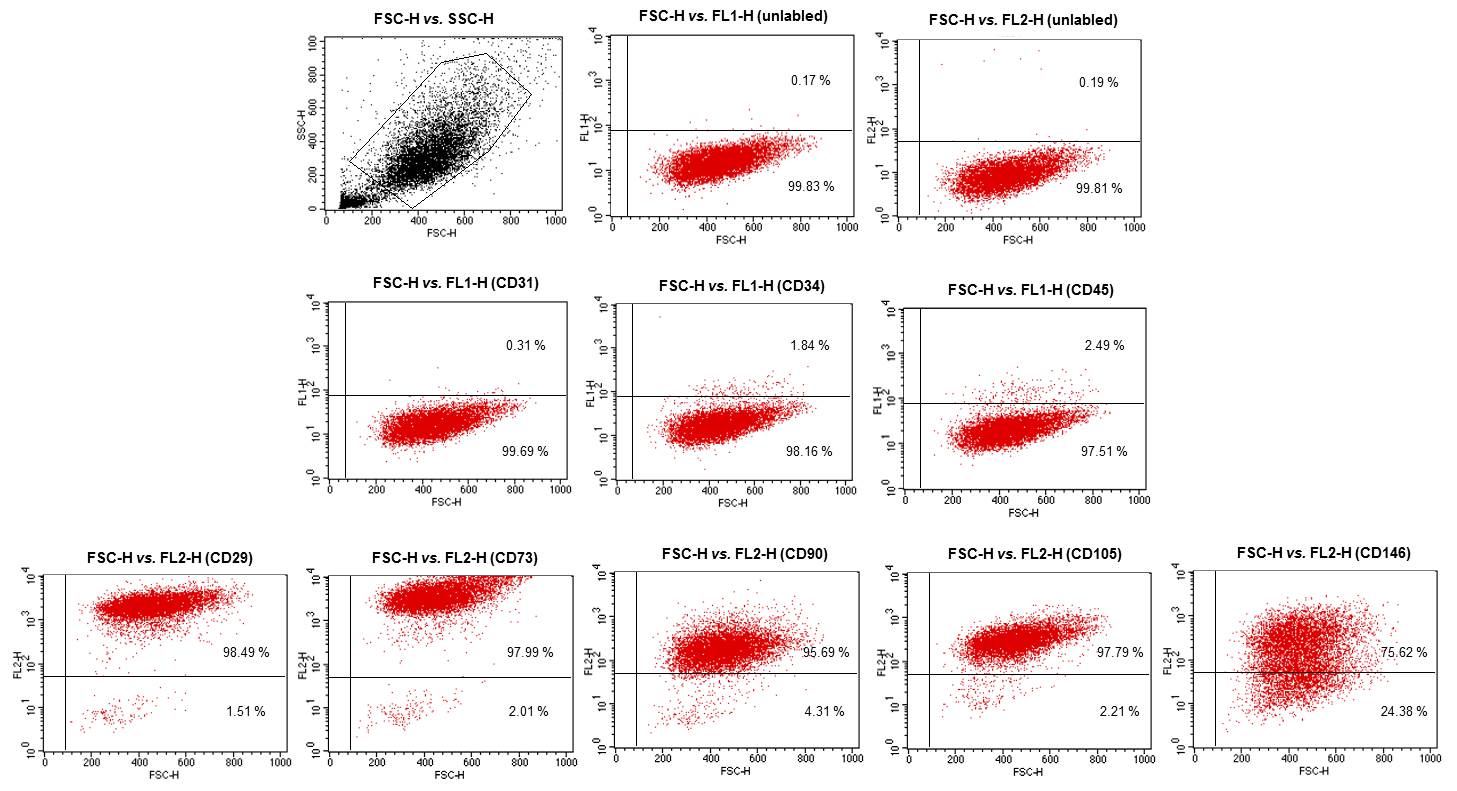


**Supplementary Figure 1: Surface marker expression of GMSCs**

Primary GMSCs were stained for mesenchymal stromal cell markers CD29, CD73, CD90, CD105 and CD146, as well as hematopoietic stem cell markers CD31, CD34 and CD45, and analysed by flow cytometry. Supplementary Figure 1 shows representative dotplots of each surface marker in comparison to the unlabled control.


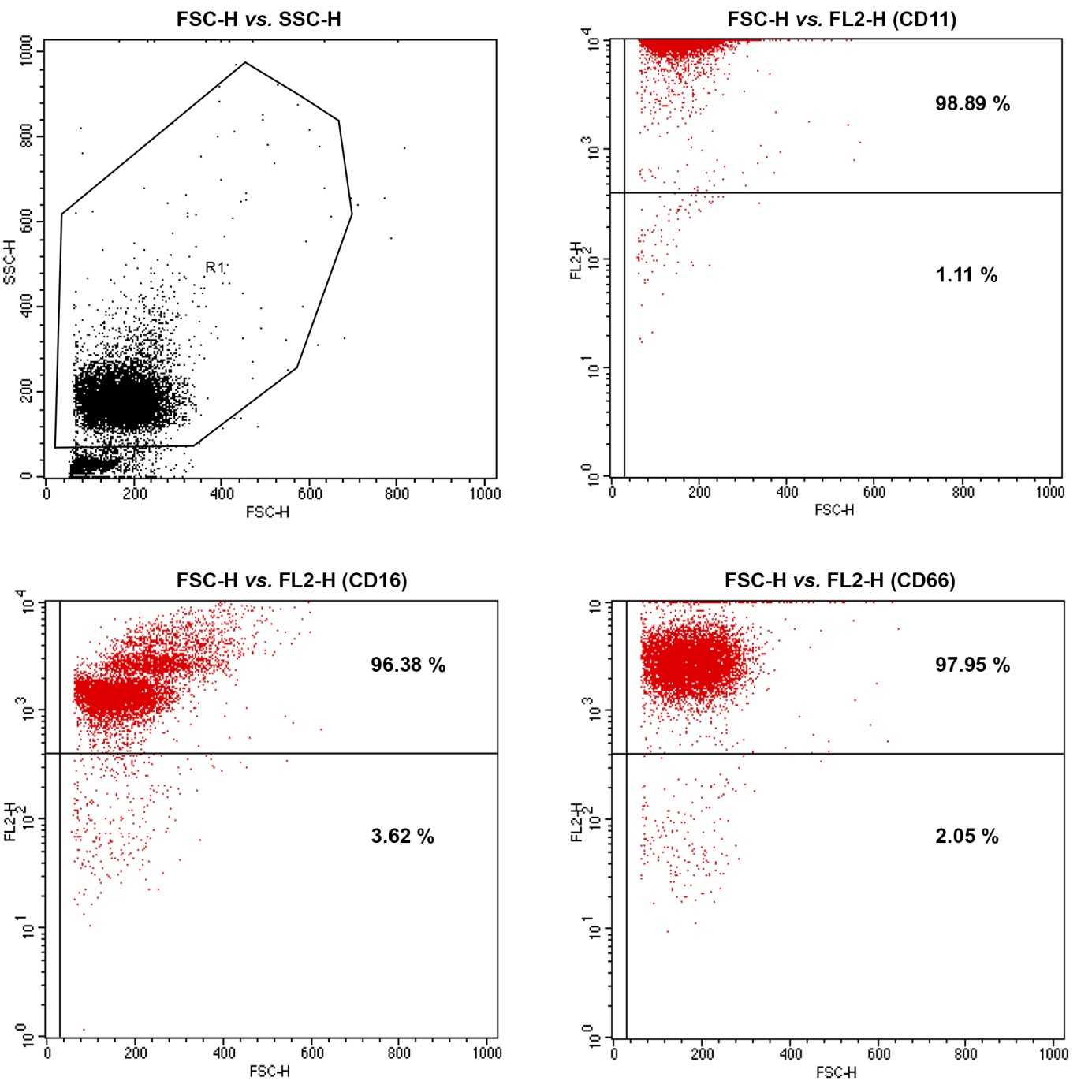


**Supplementary Figure 2: Surface marker expression of PMNs**

Primary PMNs were stained for surface markers CD11, CD16 and CD66, and analysed by flow cytometry. Supplementary Figure 2 shows representative dotplots of each surface marker.


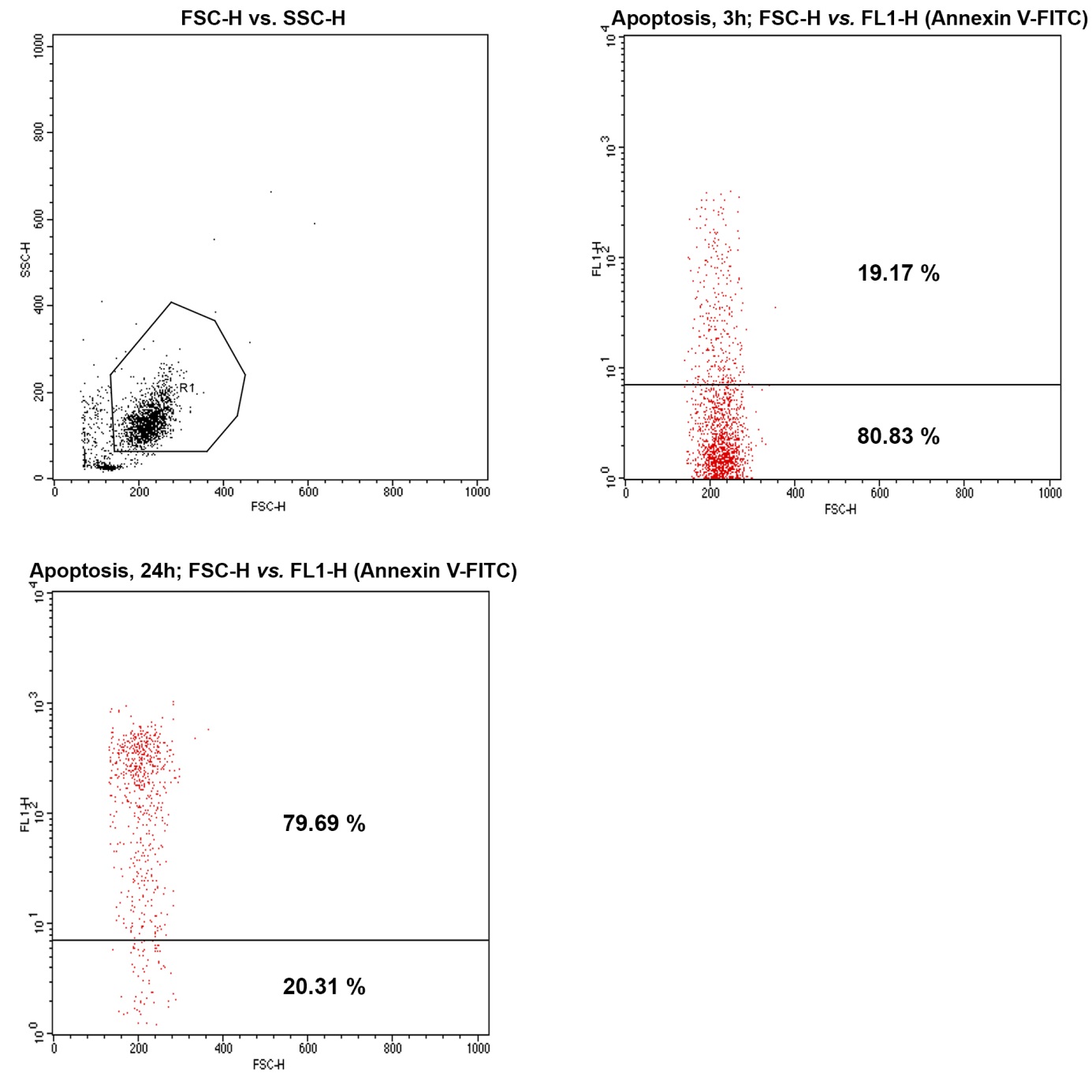


**Supplementary Figure 3: Apoptosis of PMNs after 3 and 24 hours**

Primary PMNs were incubated in monoculture with RPMI in the presence of 1µg/ml up*Pg*LPS for 3h or 24h. PMNs apoptosis was analysed by staining with Annexin V Apoptosis Detection Kit FITC and subsequent flow cytometry. Supplementary Figure 3 shows representative dotplots of Annexin V stained PMNs.


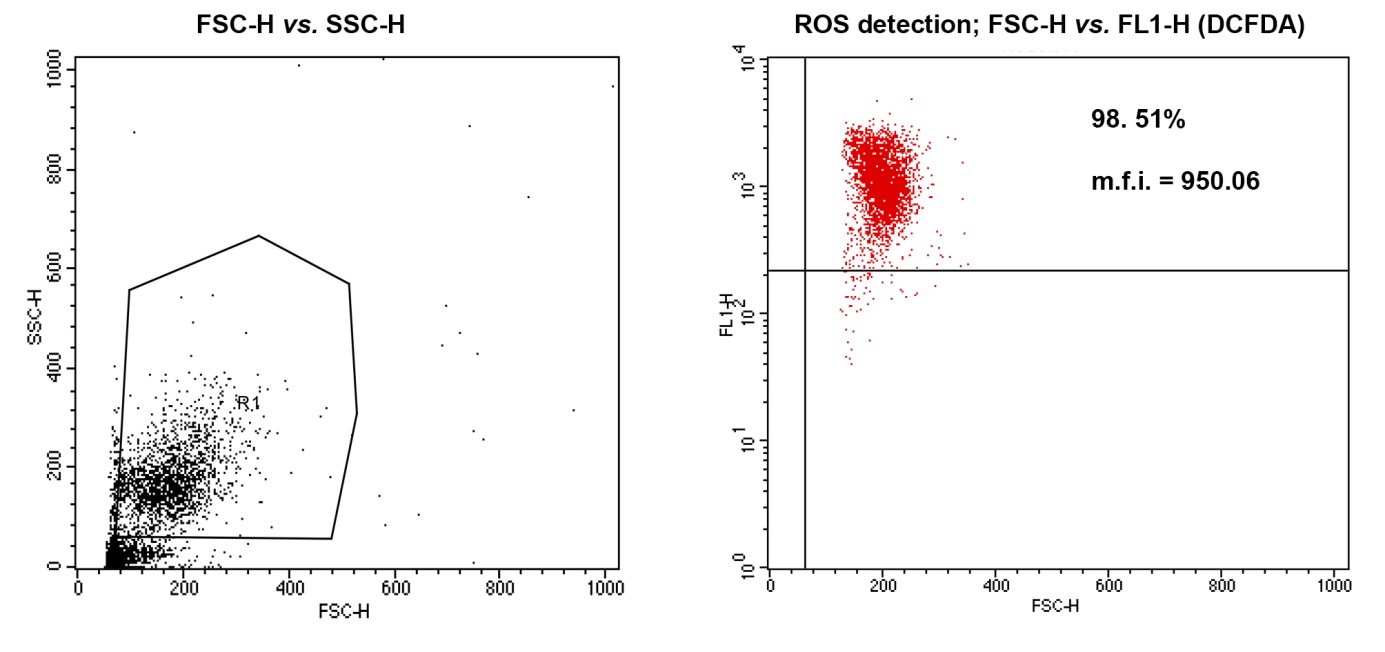


**Supplementary Figure 4: ROS production by PMNs after 15 minutes**

Primary PMNs were incubated in monoculture with RPMI in the presence of 1µg/ml up*Pg*LPS for 15 minutes. ROS production was analysed by staining PMNs with DCFDA Cellular ROS Detection Assay Kit and subsequent flow cytometry. Supplementary Figure 4 shows representative dotplots of DCFDA stained PMNs.
